# The role of amniotic epithelial cells in preterm birth: mechanisms and clinical implications

**DOI:** 10.3389/fcell.2025.1590212

**Published:** 2025-08-22

**Authors:** Lulu Meng, Jing Yang, Yijie Gao, Yiran Xie, Miaomiao Chen, Wangping Hao, Yi Luo, Ping Ru, Ling Wang, Zhiying He, Ming Liu

**Affiliations:** ^1^ Department of Obstetrics, Shanghai East Hospital, Tongji University School of Medicine, Shanghai, China; ^2^ Institute for Regenerative Medicine, Medical Innovation Center and State Key Laboratory of Cardiology, Shanghai East Hospital, School of Life Sciences and Technology, Tongji University, Shanghai, China; ^3^ Shanghai iCELL Biotechnology Co., Ltd, Shanghai, China; ^4^ Shanghai Engineering Research Center of Stem Cells Translational Medicine, Shanghai, China; ^5^ Shanghai Institute of Stem Cell Research and Clinical Translation, Shanghai, China

**Keywords:** amniotic epithelial cells (AECs), preterm birth (PTB), preterm premature rupture of membranes (PPROM), cellular senescence (CS), inflammatory responses

## Abstract

Preterm birth (PTB), defined as delivery before 37 weeks of gestation, poses a significant global health challenge. This review comprehensively examines the multifaceted role of amnion epithelial cells (AECs) in normal labor induction and preterm birth. AECs, derived from the amniotic ectoderm, exhibit paracrine effects, low immunogenicity, and non-tumorigenicity properties. They contribute to maintaining pregnancy through various aspects, such as immunomodulation, feto-maternal tolerance, and repair of placental membrane microfractures. Disruptions in AEC functions lead to preterm birth through mechanisms involving inflammation, oxidative stress, and the release of proinflammatory cytokines. This review highlights the therapeutic potentials of AECs, particularly in the context of preterm premature rupture of membranes (PPROM) and the related complications. The disruption of AECs has shown promise as a predictive biomarker for preterm birth, whereas AECs as a potential cell therapy have been shown to benefit various neonatal disorders. This review emphasizes the need for further research to fully elucidate the mechanisms underlying the role of AECs in preterm birth and to explore their clinical applications for improving pregnancy outcomes.

## 1 Introduction

Preterm birth, defined as delivery before 37 weeks of gestation, remains a global health concern due to its increasing incidence and strong association with neonatal morbidity and mortality (Chawanpaiboon et al., 2019). Preterm birth can be classified into spontaneous and iatrogenic preterm birth; the former accounts for from 70% to 80% of total occurrences of preterm births. Spontaneous preterm birth is the unexpected occurrence of threatened preterm labor, and preterm delivery before 37 weeks of pregnancy, including premature delivery and preterm premature rupture of membranes (Villar et al., 2021; Moutquin, 2003). According to the gestational age, preterm birth can be subdivided into four groups: extreme prematurity (<28 gestational weeks), severe prematurity (28–31 gestational weeks), moderate prematurity (32–33 gestational weeks), and near term (34–36 gestational weeks) (Goldenberg et al., 2008). In recent decades, the global incidence of preterm births has risen, with approximately 15 million babies born prematurely each year (Blencowe et al., 2013). Despite the unknown pathogenesis, the prevailing viewpoint is that preterm birth results from the early initiation of normal labor processes or pathological insults (Goldenberg et al., 2008). Crucially, the homeostasis of the mechanical, immune, and inflammatory properties of the fetal membrane (i.e., amniochorion membrane), a protective barrier for the developing fetus, is essential for maintaining a healthy pregnancy, while the disruption of these homeostatic processes results in fetal membrane rupture, cellular senescence, and proinflammatory milieus, which physiologically contribute to term delivery or pathologically induce preterm birth (Truong et al., 2023). During the gestation, the immunomodulatory properties of AECs contribute to feto-maternal tolerance, which is vital for preventing maternal rejection of the semi-allograft fetus and improving the outcomes of recurrent implantation failure and spontaneous abortion (Rezayat et al., 2023). In addition, AECs have been shown to release exosomes, inducing inflammatory responses in maternal uterine cells and signaling parturition (Hadley et al., 2018; Sheller et al., 2016; Monsivais et al., 2020; Kammala et al., 2023). Notably, AECs present a remarkable healing capacity to repair the microfractures of amniotic membranes through the epithelial-to-mesenchymal transition (EMT) process, which is critical for maintaining the integrity of the amniotic sac and protecting the fetus from intrauterine infections (Richardson and Menon, 2018). Another functional aspect of AECs is the ability to recognize pathogens through Toll-like receptors (TLRs) and initiate different immune responses and production of proinflammatory cytokines, such as IL-6 and IL-8, which might contribute to the onset of preterm birth (Gillaux et al., 2011). Furthermore, maternal exposure to adverse external factors like cannabis can inhibit the proliferation and migration of AECs, then subsequently leading to poor pregnancy outcomes, including preterm labor (Yao et al., 2018). Although numerous studies have investigated the association between AECs and preterm birth, the precise mechanisms underlying their interactions with immune cells and other cellular components at the maternal-fetal interface and their role in amniotic fluid homeostasis remain incompletely understood (Fathi and Miki, 2021). It is also worth noting that amniotic cells are relatively easy to collect, and since the placenta and fetal membranes are considered discarded tissues after birth, this provides a readily available and ethical source for such cells (Cappelletti et al., 2024; Naasani et al., 2022; Cocker et al., 2022). In this article, we comprehensively summarize the functional roles of AECs in normal labor induction and preterm birth and emphasize their therapeutic potential and clinical applications in neonatal disorders (Ingraldi et al., 2023).

## 2 Structural and functional characteristics of AECs

During mammalian early embryonic development, the zygote undergoes cleavage to form a blastocyst, consisting of an inner cell mass (ICM) that further differentiates either into the primitive endoderm and the pluripotent epiblast and an outer layer of trophectoderm that will form trophoblasts and eventually develop into the placenta (Deglincert et al., 2016). As an appendage of the fetus, the placenta transiently formed during pregnancy facilitates the exchange of nutrients, oxygen, and waste between the mother and fetus (Jansson, 2016). This vital extraembryonic tissue comprises maternal-derived decidua and fetal membranes fusing the outer chorion and inner amnion. The chorionic villi, primarily composed of fetal blood vessels and trophoblasts, can maximize the contact area with maternal blood and facilitate fetal-maternal exchange (Gude et al., 2004). Amnion, derived from the pluripotent epiblast of the blastocyst, forms a cavity filled with amniotic fluid that provides mechanical protection for the enclosed fetus (Nakamura et al., 2016). Structurally, the amniotic membrane comprises five layers, including AECs, basement membrane, compact layer, fibroblast layer, and intermediate spongy layer, exhibiting multiple biological functions (Mamede et al., 2012). The innermost epithelium is a protective barrier, while the basement membrane provides structural support and facilitates cell attachment (Ingraldi et al., 2023). As the innermost monolayer of amnion, amnion epithelial cells (AECs) derived from amniotic ectoderm directly contact with the amniotic fluid and possess remarkable biological characteristics, including low immunogenicity, non-tumorigenicity, as well as paracrine effects, which have been widely investigated and applied in regenerative medicine (Akle et al., 1981; Qiu et al., 2020). The compact stromal layer is rich in collagen, fibronectin, and other extracellular matrix (ECM) components, which are crucial for maintaining the integrity and mechanical framework of the amnion (Richardson and Menon, 2022). Moreover, the fibroblast layer plays a key role in tissue repair and regeneration, and the spongy layer contributes to the overall elasticity and resilience of the amnion. During pregnancy, the amnion is a physical barrier to isolate various external infections and inflammatory risk factors and actively responds to local damages, such as reactive oxygen species (ROS) (Truong et al., 2023). Amnion also contributes to maintaining ECM homeostasis through collagen remodeling, which is crucial for membrane integrity during pregnancy (Richardson et al., 2020a).

As the two predominant cellular components of amnion, AECs and amniotic mesenchymal cells (AMCs) have different embryological origins. The AECs, derived from the epiblast, form a continuous monolayer in direct contact with the amniotic fluid. The AMCs possibly originate from the later-onset extraembryonic mesoderm in the stromal layer (Silini et al., 2020). It should be noted that while AECs form a monolayer at the surface of the amnion, the underlying majority cell population comprises amniotic mesenchymal stromal cells. Regarding their biological function, AECs can migrate into wounded tissue and produce several key growth factors, bioactive cytokines, and exosomes, triggering endogenous tissue regeneration (Zhang and Lai, 2020; Nouri et al., 2018). Furthermore, transplantation of AECs into mouse models of chemotherapy-induced primary ovarian insufficiency via the tail vein or intraperitoneal cavity can partially restore ovarian function, mainly attributed to their paracrine VEGF-mediated angiogenic effects (Zhang et al., 2017; Yao et al., 2016).

On the other hand, the paracrine effects of AECs also contribute to their anti-fibrosis by secreting soluble matrix metalloproteinase (MMP)-2 and MMP-9, which are also responsible for ECM remodeling (Alhomrani et al., 2017). In addition, exosomes or extracellular vehicles (EVs) secreted by AECs carrying a variety of molecules (protein, lipids, and nucleic acids) participate in multiple intracellular crosstalk and biological activities, including wound healing, anti-apoptosis, pro-inflammation, and tissue regeneration (Hadley et al., 2018; Sheller et al., 2016; Zhang et al., 2019; Zhao et al., 2017). Exosomes are nanometer-scale (30–150 nm) membrane vesicles containing proteins, lipids, and nucleic acids released by various types of cells through exocytosis (Hessvik and Llorente, 2018). Overall, the diverse biological functions of AECs render them potential targets for interventions in various diseases.

Recent studies on amnion membrane organ-on-chip (AM-OOC) have evidenced that AECs and AMCs maintained their viability, morphology, innate meta-state, and low production of pro-inflammatory cytokines while culturing in the applied flow rate (50 μL/h, 200 μL/h) mimicking the subtle shear stress in amniotic fluids (Kim et al., 2024). Monoculture of AECs in AM-OOC undergoes the transition via EMT and migrate, eventually either revert to their original epithelial shape or maintain their achieved mesenchymal morphology. During the process, AECs express a high ratio of vimentin: CK-18 (Richardson et al., 2020a). Oxidative stress (OS) wouldn’t affect AECs’ but prevented their migration and this process can be hindered by antioxidant N-acetyl-L-cysteine (NAC).

## 3 The contributions of AECs to normal labor induction

The initiation of term labor is a physiological sterile inflammatory process involving multiple interrelated biological events, including uterine contractions, cervical remodeling, and rupture of the fetal membrane (Christiaens et al., 2008). AECs have been demonstrated to be involved in the normal labor induction via regulating the processes such as the release of prostaglandins, EMT/MET transitions, and cellular senescence.

### 3.1 Prostaglandins drive uterine contractions and cervical ripening

Prostaglandins, one of the most classic mediators of inflammation, have been extensively investigated for decades in the context of normal labor induction and progression (Olson, 2003). Particularly, elevated prostaglandin E_2_ (PGE_2_) and prostaglandin F_2α_ (PGF_2α_) levels produced by amnion play essential roles in eliciting myometrial contractions and cervical ripening (Lee et al., 2010; Myatt and Sun, 2010). Besides, prostaglandins can accelerate the degradation of collagen and lead to the rupture of fetal membranes by enhancing the expression of MMP-2 and MMP-9 and blocking the inhibitor of MMP-1 in human decidua at term (Ulug et al., 2001). Simultaneously, PGF2α is capable of stimulating the 11β-hydroxysteroid dehydrogenase 1 (11β-HSD1), thereby promoting the conversion of cortisone to cortisol. This process leads to a significant increase in the levels of cyclooxygenase-2 (COX-2), the rate-limiting enzyme in prostaglandin production, shortly before parturition (Brown et al., 1998).

### 3.2 EMT/MET transitions

Accumulating evidence suggests that as gestation processes, AECs and AMCs undergo interconversion, including transforming growth factor-β (TGF-β)-mediated EMT and progesterone (P4)-induced mesenchymal-epithelial transition (MET), which may serve as a novel trigger for parturition (Janzen et al., 2017; de Castro Silva et al., 2020; Richardson et al., 2020b). The EMT process in fetal membranes is essential for tissue remodeling, embryo implantation, placental development, and response to stress during pregnancy. Meanwhile, the irreversible EMT accompanied by accumulated AMCs are hallmarks of the amnion at parturition (Richardson et al., 2020b).

### 3.3 Cellular senescence

In addition to EMT in the amnion, the senescence of amniotic cells and the release of the senescence-associated secretory phenotype (SASP) are other factors contributing to the proinflammatory state of the fetal membrane (de Castro Silva et al., 2020). In general, the senescence of amniotic cells is physiological and synchronized with the maturation of the fetus at term. Accumulation of reactive oxygen species (ROS) results in the telomere alterations of amniotic cells by activating the stress signal p38 mitogen-activated protein kinase activation (MAPK), which accelerates cellular senescence (Menon and Papaconstantinou, 2016). The senescent amniotic cells also exhibit SASP and secrete proinflammatory cytokines, chemokines, and other signaling molecules that trigger labor (Menon et al., 2016). Moreover, the surplus generation of ROS in the full-term uterine cavity can lead to oxidative stress (OS), which can increase the endogenous production of the EMT inducer TGF-β or weaken the MET through functional inhibition of the progesterone receptor membrane components (PGRMCs) (Richardson et al., 2020b).

### 3.4 Inflammatory signaling

As another part of sterile inflammatory signals, damage-associated molecular patterns (DAMPs) are characterized by the translocation of cell-free fetal telomere fragments (cffTF) and high mobility group box (HMGB) 1 from the nucleus to the cytosol, triggering the transformation of uterine tissue to a pro-labor phenotype (Kobayashi, 2012; Stephen et al., 2015).

Apart from endocrine signaling, paracrine signaling via extracellular vesicles (e.g., exosomes) may also contribute to feto-maternal communication and serve as a component of the biological clock indicating the onset of labor (Hadley et al., 2018; Menon, 2019). It is worth noting that senescent AECs at term can release exosomes containing proinflammatory factors (e.g., SASP and p38MAPK) into the amniotic fluid and subsequently access maternal tissue via local and systemic pathways (Sheller et al., 2016). These inflammatory signals propagated through AECs-derived exosome trafficking, signaling readiness for parturition. Nevertheless, the initiation of the inflammation process in the amnion remains to be further investigated.

## 4 The involvement of AECs in preterm birth

**FIGURE 1:**
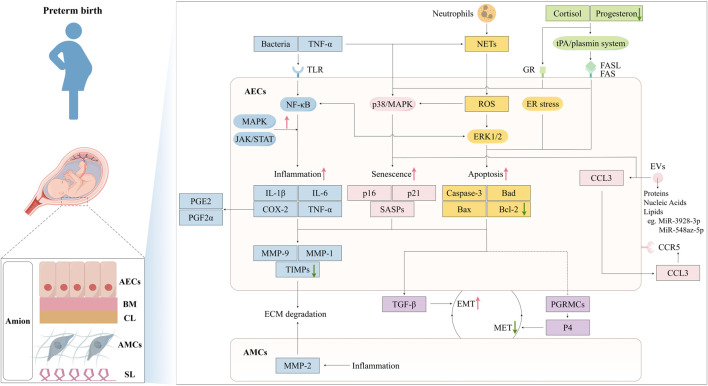
Mechanisms of Amniotic Epithelial Cells in Preterm Birth Pathogenesis. The amniotic membrane consists of five layers: amniotic epithelial cells, basement membrane, compact layer, amniotic mesenchymal cells, spongy layer. Bacterial infection and TNF-α act on TLRs on AECs, activating the NF-κB pathway and triggering an inflammatory response, leading to the production of inflammatory factors: IL-1β, IL-6, TNF-α, and COX-2. These inflammatory factors regulate chemokines MMPs and TIMPs, resulting in ECM degradation. Meanwhile, COX-2 promotes the secretion of PGE2 and PGF2α, causing uterine myometrial contractions and cervical maturation. The MAPK and JAK/STAT pathways amplify this inflammatory effect. TNF-α can also induce cell senescence through the activation of p38/MAPK, leading to the secretion of SASPs. EVs containing proteins, nucleic acids, and lipids. For instance, the overexpression of miR-548az-5p enhancing SASPs, while miR-3928-3p negatively regulates CCL3 through the CCL3-CCR5 axis and induces labor initiation signals. ROS generated by neutrophil-derived NETs can induce cellular senescence via the p38/MAPK pathway or lead to apoptosis through the ERK1/2 pathway. Upregulated cortisol and downregulated progesterone can activate the tPA/plasmin system, causing Fas-mediated apoptosis or induce cellular senescence through GR. Inflammatory factors, cellular senescence, and apoptosis can all enhance EMT, weaken MET, reduce amniotic membrane stability, and induce preterm labor. This figure was created by Figdraw.

Vaginal microorganisms influence amniotic epithelial cells mainly by ascending into the upper reproductive tract and triggering AECs (Kacerovsky et al., 2015). A healthy vaginal microbiome—dominated by *Lactobacillus* species—helps prevent this chain of events, whereas dysbiosis (such as bacterial vaginosis) increases the chance of ascending infection (Tang et al., 2024; Baldwin et al., 2015). As summarized in Figure 1, dysregulation of AEC function—through senescence, apoptosis, inflammation, extracellular matrix (ECM) degradation, and hormonal imbalance—can precipitate preterm birth (PTB). This review explores these interconnected pathways and their contributions to PTB (Table 1).

**TABLE 1:** The role of AECs in normal labor induction vs. preterm birth.

Category	AECs in normal labor induction	AECs in preterm birth	Key similarities and differences
Mechanisms	➢ EMT/MET transitions (regulated by TGF-β, progesterone) ➢ Cellular senescence (SASP release as a physiological signal) ➢ Prostaglandins (PGE_2_, PGF_2_α) drive uterine contractions and cervical ripening	➢ Pathological inflammation (NF-κB/MAPK activation) ➢ Excessive EMT leads to membrane fragility ➢ Apoptosis (caspase pathways) ➢ MMPs overexpression causes ECM degradation	Similarities: Both involve EMT, inflammation, and senescence Differences: PTB mechanisms are dysregulated (e.g., excessive MMPs, apoptosis)
Related Factors	➢ Physiological ROS, hormones (cortisol, progesterone) ➢ Exosome-mediated feto-maternal communication ➢ Telomere fragments (cffTF) as DAMPs	➢ Infections, oxidative stress ➢ Hormonal imbalance (low progesterone, high cortisol) ➢ TLR activation (e.g., TLR4-induced apoptosis) ➢ NETs promote apoptosis	Similarities: Both influenced by hormones and oxidative stress Differences: PTB involves external triggers (e.g., infection, drugs)
Key Molecules	➢ MMP-9 (controlled ECM remodeling) ➢ TGF-β, PGRMCs ➢ SASP factors (IL-6, IL-8)	➢ MMP-1/3/9 (excessive degradation) ➢ Pro-inflammatory cytokines (TNF-α, IL-1β) ➢ miR-548az-5p (senescence induction)	Similarities: Shared MMPs and cytokines Differences: PTB shows amplified/aberrant expression
Signaling Pathways	➢ TGF-β-mediated EMT ➢ PGF_2_α-11β-HSD1-COX-2 pathway (promotes prostaglandin synthesis) ➢ Exosome-mediated SASP factor transfer (sterile inflammation)	➢ TLRs-NF-κB pro-inflammatory pathway ➢ ASK1-inflammatory cascade (apoptosis promotion) ➢ Cortisol-GR-p38MAPK senescence pathway	Similarities: Both involve TGF-β and p38MAPK. Differences: PTB involves abnormal activation of TLRs and ASK1
References	Janzen et al. (2017), de Castro Silva et al. (2020), Richardson et al. (2020b), Menon et al. (2016), Menon (2019)	Gillaux et al. (2011), Romero et al. (2007), Vidal et al. (2022), Wang et al. (2020), Mogami et al. (2017), Dixon et al. (2018), Wang et al. (2016)	

### 4.1 Inflammatory responses

Amniotic epithelial cells (AECs) play a crucial role in the inflammatory processes that can trigger preterm labor. They produce proinflammatory cytokines, which can lead to the weakening of fetal membranes and ultimately result in preterm birth (Habelrih et al., 2024). AECs respond to maternal infections and stress by secreting a range of inflammatory mediators, including interleukin-1 beta (IL-1β), interleukin-6 (IL-6), tumor necrosis factor-alpha (TNF-α), and prostaglandin E2 (PGE2) (Padron et al., 2020). These mediators induce cellular senescence and apoptosis in AECs’ local immune responses that can further damage the amniotic membrane. The inflammatory environment at the maternal-fetal interface is characterized by immune cells such as macrophages and T cells, which interact with AECs and help regulate inflammation and tissue remodeling essential for maintaining pregnancy (Toothaker et al., 2020; Yang et al., 2019; Gomez-Lopez et al., 2022; Gomez-Lopez et al., 2019). The inflammation leads to the activation of matrix metalloproteinases (MMPs), which degrade extracellular matrix components, destabilizing the amniotic membrane and increasing the risk of rupture (Romero et al., 2007; Vidal et al., 2022). The inflammatory response in AECs is primarily regulated through the nuclear factor kappa-light-chain-enhancer of activated B cells (NF-κB) pathway. Upon stimulation by bacterial infections or inflammatory cytokines like TNF-α, NF-κB is activated, resulting in the upregulation of proinflammatory genes such as IL-1β, IL-6, and TNF-α. IL-37, produced by AECs, suppresses excessive inflammation, ECM remodeling, and apoptosis by inhibiting TNF-α, IL-1β, and IL-6 in the fetal membrane cells (Figure 1; Table 2) (Wang et al., 2020). These cytokines promote local inflammation and enhance the expression of MMPs, accelerating membrane rupture (Gillaux et al., 2011; de Castro Silva et al., 2020). Additionally, other pathways such as mitogen-activated protein kinase (MAPK) and Janus kinase/Signal Transducers and Activators of Transcription (JAK/STAT) also contribute to the inflammatory response by amplifying AEC reactions to external stimuli (Vidal et al., 2022). AECs are involved in producing extracellular vehicles (EVs), which facilitate cell communication and modulate immune responses. These vesicles can carry signaling molecules that influence immune cell behavior at the maternal-fetal interface, potentially affecting labor progression and timing (Hadley et al., 2018; Monsivais et al., 2020; Kammala et al., 2023; Radnaa et al., 2021; Shahin et al., 2021; Shepherd et al., 2021). The cargo of EVs includes proteins, lipids, and nucleic acids that play a role in various biological processes such as cell adhesion, leukocyte migration, and regulation of immune responses.

**TABLE 2:** Roles of cytokines, MMPs, and signaling pathways in preterm birth (PTB).

Component	Mechanism of action	Associated outcomes in PTB
IL-6, IL-1β, TNF-α	➢ Secreted by AECs in response to infection/inflammation ➢ Activate NF-κB pathway, promoting proinflammatory state ➢ Induce cellular senescence, apoptosis, and MMP expression	Fetal membrane weakening, increased risk of PPROM and preterm labor
IL-37	➢ Inhibits NF-κB and reduces IL-6/STAT3 signaling ➢ Suppresses excessive inflammation and ECM degradation	Potential protective role against PTB (anti-inflammatory)
CCL3	➢ Regulated by miR-3928-3p in AECs ➢ Activates CCR5 axis to induce cellular senescence	Promotes inflammatory environment and labor initiation
MMP-9	➢ Degrades collagen IV and V in amniotic membrane ECM ➢ Upregulated by IL-1β, TNF-α, and senescent AECs ➢ Expressed specifically by AECs (not AMCs)	Fetal membrane rupture, key driver of PPROM
MMP-2	➢ Degrades collagen I and III ➢ Produced by AMCs (not AECs) ➢ Less relevant to PTB compared to MMP-9	Minor role in membrane degradation
MMP-1, MMP-3	➢ Contribute to ECM remodeling and degradation ➢ Elevated in inflamed fetal membranes	Structural instability of amnion
NF-κB	➢ Central regulator of inflammation ➢ Activated by TLRs, cytokines (e.g., TNF-α) ➢ Promotes MMP expression and epithelial apoptosis in AECs	Fetal membrane inflammation and rupture
p38 MAPK	➢ Induces cellular senescence and SASP in AECs ➢ Activated by ROS and telomere fragments ➢ Senescent AECs release exosomes with p38MAPK components	Accelerated senescence, sterile inflammation, and labor initiation
TGF-β/EMT	➢ Mediates epithelial-mesenchymal transition in AECs for normal wound healing ➢ Dysregulated by inflammation, leading to excessive EMT and membrane weakening	Impaired membrane integrity and preterm rupture (e.g., PPROM)
PI3K/Akt	➢ Regulated by progesterone to inhibit MMP expression ➢ Reduced progesterone in PTB impairs PI3K/Akt signaling, leading to uncontrolled MMP activity	Uncontrolled ECM degradation and membrane instability

Activating inflammatory responses in amniotic epithelial cells involves various cytokines, signaling pathways, and immune cell interactions. Dysregulation of these processes can lead to adverse pregnancy outcomes, including preterm birth. Understanding these mechanisms is critical for developing potential therapeutic strategies to mitigate the risks associated with preterm labor.

### 4.2 MMPs regulation and ECM degradation

MMPs are a family of zinc-dependent endopeptidases that play a crucial role in the degradation and remodeling of the extracellular matrix (ECM). They are involved in various physiological processes, including tissue repair and remodeling, as well as pathological conditions such as inflammation and cancer. MMPs, such as MMP-1, MMP-3, and MMP-9, are secreted by AECs and are particularly significant in the context of PTB because they contribute to ECM degradation, which can compromise the structural integrity of the amniotic membrane (Lukes et al., 1999; Radulescu et al., 2021). AECs are essential for maintaining the amniotic membrane’s structural integrity. Under normal conditions, the ECM comprises collagen, elastin, and glycosaminoglycans, which provide mechanical strength. However, during PTB, AECs secrete elevated levels of MMPs that lead to excessive degradation of these ECM components. This overactivity can weaken the membrane and increase the risk of rupture (Xu et al., 2002). In response to inflammatory stimuli such as interleukin-1 beta (IL-1β) and tumor necrosis factor-alpha (TNF-α), AECs upregulate MMP gene expression. Specifically, MMP-9 is known for degrading collagen types IV and V, while MMP-1 targets type I and III collagen—significant amniotic membrane components. The dysregulation of MMP activity can lead to an imbalance between MMPs and their inhibitors, tissue inhibitors of metalloproteinases (TIMPs), resulting in ECM degradation and increased risk of membrane rupture. AECs can undergo EMT, losing their epithelial characteristics and gaining mesenchymal properties. This transition has been linked to the weakening of the amniotic membrane and is considered a significant factor in preterm premature rupture of membranes (PPROM) (Presicce et al., 2023). Proinflammatory cytokines can induce EMT in AECs, while inhibiting this process may help protect against PPROM (Janzen et al., 2017; Richardson et al., 2020b; Mogami et al., 2017; Menon et al., 2020; Weed et al., 2020; Kawamura et al., 2022; Menon, 2022; Wang et al., 2023; Severino M. E. et al., 2024; Severino M. E. L. et al., 2024). The regulation of MMP activity by AECs is critical for maintaining the integrity of the amniotic membrane. Dysregulation due to excessive MMP expression or reduced TIMP levels can lead to significant ECM degradation, increasing the likelihood of rupture during PTB.

### 4.3 Cellular senescence

The cellular senescence of amniotic epithelial cells is a critical factor leading to the underlying preterm birth. Cellular senescence refers to a state where cells stop dividing but remain metabolically active, secreting various proinflammatory cytokines (a phenomenon known as the senescence-associated secretory phenotype, SASP). Oxidative stress, inflammation, and microRNA regulation have been demonstrated to be involved in the senescence of AECs, which can compromise amniotic membrane integrity and promote preterm labor (Menon et al., 2020; Dutta et al., 2016; Sheller-Miller et al., 2017). However, the relevant mechanisms have not been fully illustrated, and further research is still needed.

AECs are particularly susceptible to oxidative stress (OS) and inflammatory signals, which can induce senescence. Studies have demonstrated that exposure to proinflammatory cytokines, such as TNF-α, can activate the p38MAPK pathway in AECs, increasing senescence markers and secretion of inflammatory cytokines, such as IL-6 (Dixon et al., 2018). Similar results have been observed in amniotic membranes in term labor, suggesting that similar mechanisms may be at play during preterm labor. Senescent AECs secrete inflammatory mediators such as IL-1β, IL-6, TNF-α, and others, which activate local inflammatory responses. These inflammatory cytokines further induce the degradation of extracellular matrix (ECM) components, leading to structural instability of the amniotic membrane and accelerating its premature rupture. Increased MMPs associated with senescent cells may lead to degradation of extracellular matrix components, facilitating conditions such as preterm premature rupture of membranes (PPROM). This degradation can trigger a cascade of events leading to PTB. The presence of senescent AECs contributes significantly to membrane disruption, a key event in initiating PTB (Menon et al., 2020). For instance, miR-548az-5p has been identified as a microRNA that induces senescence in AECs, contributing to labor initiation (Jing et al., 2024). Overexpression of miR-548az-5p has enhanced SASP, further contributing to the inflammatory environment associated with labor initiation (Jing et al., 2024). The accumulation of senescent cells can release proinflammatory cytokines, creating a hostile environment that may trigger preterm labor. Hsa-miR-3928-3p negatively regulates CCL3, promoting AECs senescence through the CCL3-CCR5 axis and inducing signals for labor initiation (Liu et al., 2024).

Additionally, telomere fragments released from senescent cells have been shown to induce further oxidative stress and inflammatory responses, potentially exacerbating the conditions leading to preterm birth. The studies have shown that telomere fragments released from senescent fetal cells can trigger oxidative stress responses in AECs (Phillippe, 2022; Polettini et al., 2015; Saroyo et al., 2021). These fragments are associated with the activation of p38MAPK and the senescence-associated secretory phenotype (SASP), which includes a variety of inflammatory mediators (Polettini et al., 2015; Lavu et al., 2019). The accumulation of these fragments may contribute to a sterile inflammatory response implicated in initiating labor at term or prematurely.

The involvement of cellular senescence of AECs is a critical factor in the underlying mechanisms of preterm birth. Further research into these mechanisms may offer insights into potential therapeutic strategies to prevent PTB and improve neonatal outcomes.

### 4.4 Cellular apoptosis

Apoptosis of AECs has been reported to be another underlying mechanism contributing to PTB. Research indicates that elevated cortisol levels towards the end of gestation promote the apoptosis of amniotic epithelial cells. Cortisol activates the tissue-type plasminogen activator (tPA)/plasmin system, leading to apoptosis in the epithelial cells, sparing fibroblasts. This process involves a Fas-mediated extrinsic apoptotic pathway, which is crucial for the structural remodeling of the amnion prior to rupture of membranes (ROM) at both term and preterm births (Wang et al., 2016).

Inflammation, particularly from infections, triggers apoptosis of amniotic epithelial cells through various pathways. For instance, the activation of apoptosis signal-regulating kinase 1 (ASK1) contributes to inflammation-induced preterm birth by enhancing the production of proinflammatory cytokines, thereby intensifying the inflammatory response in AECs (Yoshikawa et al., 2020). Inflammation at the maternal-fetal interface can lead to the infiltration of neutrophils, which produce neutrophil extracellular traps (NETs) (Gomez-Lopez et al., 2022; Galaz et al., 2020; Motomura et al., 2022; Gomez-Lopez et al., 2014). Compared to normal term birth, preterm birth showed increased NETs infiltration and MMP-9 expression in the amniotic membrane, and NETs promote AECs apoptosis and inhibit their proliferation through ROS and ERK1/2 pathways (Hu et al., 2023). AECs apoptosis is associated with increased MMPs expression, extracellular matrix degradation, and membrane weakening (Hu et al., 2023). The apoptotic process disrupts the integrity of the amniotic membrane and activates MMPs that degrade extracellular matrix components. The degradation of ECM further compromises amniotic membrane strength and leads to ROM, a significant precursor to preterm birth (Wang et al., 2016). Apoptotic AECs release endogenous proteins and signaling molecules that can trigger inflammation and compromise membrane integrity. Apoptosis is triggered by activating caspase family proteins (caspases), leading to cellular disintegration and membrane rupture. When AECs are exposed to stress or inflammatory cytokines, the caspase pathways are activated, triggering programmed cell death. Apoptosis is also closely associated with endoplasmic reticulum (ER) stress, where the accumulation of misfolded proteins prompts autophagy or apoptotic pathways. Amniotic epithelial cells express functional TLR5, TLR6/2, and TLR4 (Gillaux et al., 2011). Activation by TLR5 and TLR6/2 agonists produces IL-6 and IL-8, concomitantly with the activation of NF-κB signaling pathway, matrix metalloproteinase-9 induction, and PTGS2 expression. In contrast, TLR4 activation reduced amniotic epithelial cell viability and induced cell apoptosis, evidenced by an elevated Bax/Bcl-2 ratio and cleavage of caspase-3 (Gillaux et al., 2011). These data suggest specific TLR-mediated functions in human amniotic epithelial cells for initiating different responses, which may lead to preterm birth.

The interplay between cellular apoptosis and inflammation in amniotic epithelial cells significantly influences the occurrence of preterm birth. Understanding these processes is crucial for developing therapeutic strategies to prevent preterm labor and improve outcomes for affected neonates. Further research is needed to elucidate the exact pathways involved and how they can be targeted for intervention.

### 4.5 Hormonal regulations for AECs function

The function of AECs is significantly influenced by hormonal changes during pregnancy. Key hormones such as progesterone, estrogen, and cortisol play crucial roles in regulating AEC proliferation, differentiation, and stress responses. Understanding these hormonal interactions is vital, especially in PTB, where hormonal imbalances can lead to AEC dysfunction. Progesterone is essential for maintaining pregnancy and influences AEC behavior. It regulates AEC proliferation and differentiation through its receptor (PR), and low levels of progesterone can lead to a decline in AEC proliferative capacity, resulting in structural instability of the amniotic membrane (Kumar and Magon, 2012).

Additionally, progesterone modulates the PI3K/Akt signaling pathway to inhibit matrix metalloproteinase (MMP) expression, thereby protecting the amniotic membrane from excessive degradation (Locksmith et al., 2001; Allen et al., 2014). Estrogen, produced by both the ovaries and placenta, supports various aspects of pregnancy, including the development of fetal tissues and the modulation of immune responses (Robinson and Klein, 2012). Its role in AEC function is less direct but contributes to overall hormonal balance during pregnancy. Elevated cortisol levels can induce senescence or apoptosis in AECs by binding to glucocorticoid receptors (GR), activating downstream signaling pathways that promote cellular aging. Research indicates that cortisol may enhance the expression of senescence-associated secretory phenotype (SASP) factors via activation of the p38 MAPK pathway, which can contribute to membrane rupture (Martin et al., 2019). This mechanism highlights how stress responses mediated by cortisol can negatively impact AEC integrity. In cases of PTB, hormonal imbalances—such as elevated cortisol and reduced progesterone—can lead to significant dysfunction in AECs. Elevated cortisol may cause premature senescence or apoptosis in these cells, while decreased progesterone impairs their ability to maintain membrane integrity (Kumar and Magon, 2012). This dysfunction can compromise the amniotic membrane’s structural stability, increasing the risk of preterm labor.

### 4.6 Mechanobiology of AECs and the fetal membrane

The mechanobiology of AECs and the fetal membrane is a critical factor in understanding preterm birth, particularly through the lens of mechanical stress, membrane tension, and uterine stretch. Recent studies have elucidated the structural and functional changes in the fetal membranes that contribute to preterm premature rupture of the membranes (pPROM) (Richardson et al., 2017). For instance, microfractures in the amnion, characterized by epithelial shedding, basement membrane damage, and tunnels through the extracellular matrix (ECM), have been identified as significant contributors to membrane weakening (Menon and Richardson, 2017). These microfractures are more pronounced in preterm conditions, with morphometric measures (width and depth) higher in term labor compared to preterm cases, suggesting a predisposition to rupture in pPROM (Menon et al., 2019). Oxidative stress and inflammation further exacerbate this process by inducing premature senescence, preventing normal remodeling and leading to membrane rupture. The role of mechanical forces, such as uterine stretch, is also evident, as it can alter AEC function and ECM homeostasis, contributing to the initiation of labor signals.

AECs are multifaceted cells that play a critical role in pregnancy. Compared with other placental cell populations, AECs exhibit unique immunomodulatory and reparative functions that differentiate them from chorionic trophoblasts and decidual stromal cells (DSCs). AECs—derived from the embryonic epiblast—retain partial pluripotency and secrete anti‐inflammatory cytokines such as IL‐10 and TGF‐β, which contribute to fetal immune tolerance and maintenance of amniotic membrane integrity (Miki, 2018; Murphy and Atala, 2013). In contrast, chorionic trophoblasts (including syncytiotrophoblasts and extravillous trophoblasts) primarily mediate maternal–fetal interface remodeling through invasive behavior and hormone production, playing a pivotal role in placental anchoring and nutrient exchange (Ma et al., 2015). DSCs, which arise from maternal endometrial stromal cells, create a decidual microenvironment characterized by prostaglandin and cytokine secretion that regulates local immune cell populations and supports embryo implantation (Gellersen and Brosens, 2014). Therefore, AECs are distinguished by their dual capacity for anti‐inflammatory signaling and, conversely, SASP‐driven extracellular matrix remodeling, underscoring their unique relevance in the pathogenesis of preterm birth.

## 5 Clinical application prospects of AECs

Preterm birth remains a major health challenge worldwide, as premature infants have an inherently higher risk of severe neonatal morbidity, including neurological, respiratory, and cardiovascular complications (Raju et al., 2017). Despite increasing research into the pathogenesis of preterm birth, there remains a lack of diagnostic tools that can accurately predict the onset of spontaneous preterm birth or PPROM. Drawing on this background, the Biomarkers Groups of PREBIC (The Preterm Birth International Collaborative) developed several technologies for predicting preterm birth, among which cell-free RNA in the amniotic fluid, proteomic analysis, and fetal membrane cells in the maternal circulation have gained extensive research attention (Lamont et al., 2020). A recent study revealed that specific markers in AECs are linked to PPROM, suggesting that these cells could be utilized as predictive biomarkers for preterm birth in the clinic (Mikkelsen et al., 2023). Specifically, RNA sequencing data identified 73 upregulated transcripts in the fetal membrane cells. After evaluation by immunohistochemistry, only MUC16 (also named CA125) was exclusively expressed in the AECs. As a well-known biomarker for detecting epithelial ovarian cancer, circulating CA125 has never been investigated as a predictor before the onset of preterm birth, although its expression increased during pregnancy (Han et al., 2012). However, elevated CA125 in the amnion fluid is associated with intra-amniotic inflammation, indicating imminent delivery in patients diagnosed with PPROM (Seong, 2016). AECs-specific biomarkers or AECs-derived exosomes associated with inflammatory processes make this type of fetal membrane cell an emerging predictor of preterm birth.

On the other hand, AECs possess unique properties such as immunomodulation and reparative effects, reflecting their therapeutic potential for PPROM and preterm birth complications. Recent studies indicated that fetal macrophages assist in the repair of ruptured amnion by promoting the TGF-β-mediated EMT process in AECs (Mogami et al., 2017; Kawamura et al., 2022; Hao et al., 2025). It has been demonstrated that human amniotic epithelial cells (hAECs) exert multiple immunosuppressive activities, such as inhibiting T and B cell proliferation, suppressing inflammatory properties of monocytes, macrophages, and natural killer cells, promoting induction of cells with regulatory functions such as Treg and the anti-inflammatory M2 macrophages (Magatti et al., 2008; Banas et al., 2008; Wolbank et al., 2007; Morandi et al., 2023). Additionally, several studies showed that hAECs therapeutic potentials because of their underlying properties in low immunogenicity, anti-fibrosis, and tissue repair. These features make hAECs a very attractive cellular source in regenerative medicine and transplantology (Zhang and Lai, 2020; Lim et al., 2024). While hAECs are promising for cell-based therapies, exhibit poorly defined proliferative dynamics. Our study first delineates gestational age- and passage-dependent transcriptional landscapes of AECs through scRNA-seq analysis (Hao et al., 2025). Term-derived AECs initiate epithelial-mesenchymal plasticity (EMP) via rapid partial EMT (pEMT) upon *in vitro* culture, enabling transient proliferation before senescence-driven EMT progression. Leveraging this mechanism, we established an innovative 3D culture platform combining SB431542 (TGF-β inhibitor) with microcarriers, achieving 50-fold expansion while preserving EMP. This scalable system yields therapeutic-grade AECs from a single donor sufficient for 50 patients, resolving critical clinical translation barriers. Our findings bridge developmental biology insights with translational innovation in regenerative medicine (Hao et al., 2025). In addition, an *in vitro* study using the amniotic pore culture technique confirmed the efficacy of human amniotic epithelial stem cells (hAESCs) in accelerating the healing of preterm ruptured fetal membranes (Kang et al., 2022; Lee et al., 2020).

Furthermore, AECs with high safety and wider access hold great promise as a novel cell therapy for various diseases unique to premature infants, including bronchopulmonary dysplasia (BPD), hypoxic-ischemic encephalopathy, and necrotizing enterocolitis (O'Connell et al., 2021; Baker et al., 2019; Davidson et al., 2022; Lim et al., 2018). As the first and most investigated application, AECs exerting anti-inflammatory and anti-fibrotic effects for BPD have progressed to clinical trials (Baker et al., 2019; Malhotra et al., 2020) (Table 3). Further investigation has shown that preterm AECs displayed limited regenerative properties compared to term AECs in the bleomycin mouse model of acute lung injury (Lim et al., 2013).

**TABLE 3:** Summarized registrations of clinical trials utilizing human amniotic epithelial cells as biological interventions in neonatal diseases.

Registration number	Disease	Nation	Phase
ACTRN12618000920291 (Baker et al., 2019)	Bronchopulmonary Dysplasia, Extremely Preterm Birth	Australia	I
ACTRN12614000174684 (Lim et al., 2018; Malhotra et al., 2020)	Bronchopulmonary dysplasia	Australia	Ib
NCT03107975	Spastic Cerebral Palsy	China	I

Despite the promising therapeutic potential of AECs, several ethical, logistical, and regulatory challenges must be addressed before their routine clinical application. Ethically, AECs are considered a non-controversial cell source, as they are derived from term placentas usually discarded after delivery, with donor consent mitigating concerns related to embryonic or fetal tissue use (Miki et al., 2005). Nonetheless, rigorous ethical oversight is essential to ensure voluntary and informed maternal consent, especially when cells are to be used for allogeneic therapies. Logistically, the standardization of AEC isolation, cryopreservation, and large-scale expansion remains a significant hurdle due to donor variability, low proliferation rates, and loss of stemness with prolonged culture (Akle et al., 1981). Regulatory barriers further complicate translation, as AEC-based therapies fall under advanced therapy medicinal products (ATMPs) in many jurisdictions, necessitating compliance with Good Manufacturing Practice (GMP), extensive safety testing, and long-term follow-up (Viswanathan et al., 2013). Harmonizing international guidelines and establishing centralized biobanks may facilitate broader access and ensure consistency in cell quality and therapeutic efficacy. Addressing these multifaceted issues is critical to transitioning AECs from bench to bedside in the prevention and treatment of preterm birth.

Taken together, AECs provide a promising avenue for addressing the prediction and treatment of preterm birth. The future direction of preterm diagnosis lies in combining biomarkers differentially expressed by AECs with non-invasive maternal blood sampling to assess the intra-amniotic inflammatory status comprehensively. Regarding therapeutic aspects, AECs have the advantages of immunomodulatory properties, regenerative capacity, non-tumorigenicity, and low immunogenicity compared with other stem cell therapies (Miki, 2018; Rosner et al., 2012; Muttini et al., 2018; Pratama et al., 2011). Meanwhile, there are some drawbacks of AECs therapy (Pratama et al., 2011; Chang et al., 2017). Firstly, the proliferation capacity of hAECs is restricted due to the lack of telomerase activity, and they are prone to senescence and EMT during *in vitro* culture. Secondly, the biological characteristics of hAECs are intricate, with their differentiation potential not yet fully understood, and the expression of cell markers is variable, posing difficulties for cell identification and quality control. In terms of clinical application, expanding hAECs on a large scale is problematic, with no standardized culture system established, and there is a risk of immune rejection. Additionally, regulatory and ethical issues, such as quality control and ethical controversies, need to be considered. Other limitations include the possibility of teratoma formation, short-term therapeutic effects, and unclear mechanisms of action. Despite these issues, hAEC-based therapy exhibits broad potential in disease treatment. Future research should focus on addressing these challenges to facilitate the clinical application of hAECs.

## 6 Conclusions and future challenges

The conclusions drawn from the comprehensive review of the current literature on amniotic epithelial cells (AECs) underscore their critical role in the complex biological processes surrounding pregnancy and preterm birth. AECs contribute significantly to maintaining pregnancy homeostasis through their immunomodulatory functions, participation in the EMT, and ability to release exosomes that influence maternal uterine cells. The evidence suggests that disruptions in AEC function, whether due to external factors, inflammatory responses, or cellular senescence, can lead to preterm birth. Furthermore, AECs exhibit potential as biomarkers for predicting preterm birth and as a therapeutic modality in regenerative medicine, highlighting their reparative and immunomodulatory capabilities.

Despite promising insights into the role of AECs, several challenges and knowledge gaps remain to be addressed. There is a need for a more profound understanding of the molecular mechanisms underlying AEC function, mainly how they interact with other cell types in the amniotic environment and contribute to preterm birth. While AECs show potential as predictive biomarkers, further research is required to identify, validate, and clinically implement these markers to improve the prediction of preterm birth. The development of standardized protocols for using AECs in therapeutic applications is essential to ensure consistency and reliability in treatment outcomes. AEC-based therapies’ long-term safety and efficacy must be rigorously evaluated, especially considering the variable efficacy observed in different pregnancy conditions. Exploring the synergistic effects of AEC therapies with other treatment modalities could enhance their overall impact on preterm birth prevention and treatment. Designing and conducting well-structured clinical trials to test the efficacy of AEC-based interventions is crucial for translating laboratory findings into clinical practice. As with any cellular therapy, ethical considerations surrounding the use of AECs must be carefully addressed, particularly in the context of stem cell research and therapy.

Addressing these challenges will be crucial for harnessing the full potential of AECs in improving pregnancy outcomes and reducing the incidence of preterm birth. Continued research, interdisciplinary collaboration, and innovative clinical trial designs will be instrumental in overcoming these hurdles and advancing the field.
